# Investigating the Mechanical Behavior and Energy Absorption Characteristics of Empty and Foam-Filled Glass/Epoxy Composite Sections under Lateral Indentation

**DOI:** 10.3390/ma17153847

**Published:** 2024-08-03

**Authors:** Seyedahmad Taghizadeh, Abbas Niknejad, Lorenzo Maccioni, Franco Concli

**Affiliations:** 1 Faculty of Engineering, Free University of Bozen-Bolzano, Piazza Università 5, 39100 Bolzano, Italy; seyedahmad.taghizadeh@student.unibz.it (S.T.); lorenzo.maccioni@unibz.it (L.M.); 2 Mechanical Engineering Department, Yasouj University, Yasouj 75918-74934, Iran; abbas.niknejad@yu.ac.ir

**Keywords:** composite tube, lateral indentation, energy absorption, lightweight design, polyurethane foam

## Abstract

In this study, the crashworthiness behavior and energy absorption capacity of composite tubes under lateral indentation by steel rods aligned parallel to the specimen axis are investigated using experimental methods. Key parameters such as tube diameter, length, wall thickness, and indenter diameter are systematically examined and compared. Additionally, the influence of polyurethane foam fillers on damage modes and energy absorption capacity is rigorously analyzed. Contrary to conventional findings, smaller diameter specimens filled with foam demonstrate superior energy absorption compared to their larger counterparts, primarily due to enhanced compression of the foam volume. Experimental results reveal a complex interplay of damage mechanisms in composite specimens, including matrix cracking, fiber breakage, foam crushing, foam densification, foam fracture, and debonding of composite layers, all contributing to enhanced energy absorption. Increased tube thickness, length, and indenter diameter, alongside decreased tube diameter, are correlated with higher contact forces and improved energy absorption. Smoother shell fractures are promoted, and overall energy absorption capabilities are enhanced by the presence of foam fillers. This investigation provides valuable insights into the structural response and crashworthiness of composite tubes, which is essential for optimizing their design across various engineering applications.

## 1. Introduction

In the realm of structural engineering and materials science, extensive investigations spanning the past three decades have highlighted the exceptional performance of composite materials in crashworthiness applications. Their adaptability in size, shape, and fabrication techniques empowers researchers and industries to achieve targeted mechanical performance, rendering them highly suitable across diverse applications [1,2,3]. Composite materials not only satisfy rigorous design criteria across various sectors but also address customer demands for safer, more efficient structures with reduced fuel consumption and enhanced payload capacities. Recent research has notably concentrated on the axial and lateral compression behaviors of metallic and composite structures and columns [4,5]. Composite tubes have garnered significant attention due to their exceptional energy absorption capabilities, lightweight characteristics, and potential for bolstering overall structural crashworthiness. Studies in this area highlight the pivotal role of composite tubes in advancing safety and performance standards across multiple industries. For example, Jafar Rouzegar et al. [6] investigated the flattening process of composite–metal tubes through experimental analysis. Their study involved the lateral compression of empty and foam-filled specimens to ascertain specific absorbed energy (SAE) and construct load–displacement curves. Factors such as tube length, foam filler type and density, and adhesive properties were evaluated for their influence on energy absorption. Their findings revealed linear correlations between tube length, foam density, and energy absorption, with foam fillers notably enhancing absorbed energy. Composite tubes demonstrated superior energy dissipation capabilities compared to metal tubes, albeit with less regular deformation patterns. In addition, hybrid composite–metal tubes, leveraging the strengths of both materials, achieved notable crush force efficiencies (CFE), peaking at 0.76. Energy absorption outcomes were significantly affected by factors such as metal tube fracture, foam filler characteristics, and delamination within composite layers. Yantao Gao et al. [7] analyzed the mechanical responses and failure behaviors of advanced composite tubes designed for nuclear applications, employing acoustic emission (AE), imaging techniques, and finite element modeling (FEM). Their research identified progressive damage behaviors akin to metals, with fractures primarily initiated along symmetric longitudinal lines. Additionally, monitoring through AE signals effectively tracked damage evolution, although the load-bearing capacity of the tubes could be considerably compromised due to delamination, large crack formation, or fiber bundle fractures. Research by Xiaoxu Wang et al. [8] investigated three-dimensional braided composite tubes with varied diameter-to-thickness (D/t) ratios (60, 30, and 20) through lateral compression tests. Their findings highlighted increased lateral failure loads with decreasing D/t ratios. Thin-walled tubes exhibited sudden failure modes, while thicker-walled variants underwent progressive damage following slight load reductions. Primary failure mechanisms included fiber extraction, fracture, matrix cracking, and debonding between fibers and matrix material. Furthermore, their study contributes significant insights into understanding how D/t ratios influence the mechanical behavior and failure modes of composite tubes, which are crucial for optimizing their design and practical application in engineering contexts. Azadeh Jalali and Ali Massumi [9] examined composite diagrid structures under lateral seismic loads, focusing on inner-braced and inner-moment frame cores in 36-story diagrids with varying diagonal angles. Their analysis, employing response spectrum and nonlinear static methods, revealed that increasing diagonal brace slopes diminished lateral strength in both core types. Diagrids with 50.19° diagonal brace angles and inner braced cores exhibited heightened strength but displayed brittleness under lateral loads, prone to diagonal brace buckling. Recommendations included enhancing diagrid ductility through the incorporation of buckling restrained elements and advocating further nonlinear dynamic analyses to validate findings across diverse aspect ratios. Tamer A. Sebaey et al. [10] explored the effects of aging duration and temperature on carbon fiber-reinforced plastic (CFRP) rectangular tubes, polyurethane foam, and foam-filled CFRP tubes during lateral crushing tests. Their study demonstrated that elevated temperatures and prolonged exposure times led to diminished peak and average crushing loads, attributable to matrix cracking and fiber–matrix debonding. Conversely, polyurethane foam displayed enhanced strength and energy absorption under compression load with aging, while foam-filled CFRP tubes exhibited sustained energy absorption and reduced sensitivity to thermal aging, maintaining higher crush force efficiencies. Mu-Xuan Tao et al. [11] investigated the influence of concrete cracking on redistribution factors in composite frame structures subjected to lateral loads, utilizing shell-solid models. They proposed formulas based on extensive model analysis, highlighting reduced load capacities in beams and negative moment areas. Recommendations included utilizing average von Mises stress in the lower flange’s mid-layer as a yield criterion, emphasizing critical parameters such as column–beam stiffness ratios and beam spans for optimizing structural design against concrete cracking effects. Bing Zhang et al. [12] conducted axial compression and cyclic lateral loading tests on elliptical concrete-filled FRP tubes embedded with H-shaped steel. Their findings underscored significant energy dissipation capabilities due to effective FRP confinement that prevented local buckling of H-shaped steel. Minimal impact on ductility and energy dissipation was observed with varying elliptical aspect ratios, while increasing FRP thickness marginally improved peak load and stiffness degradation. Zheng Li et al. [13] investigated the lateral performance of low-rise aluminum alloy frames filled with composite wallboards, quantifying hysteretic performance and lateral load-resisting capacities. Their study provided technical insights into the application of low-rise aluminum alloy frames incorporating composite wallboards based on empirical findings. Xia Yang et al. [14] proposed a design integrating mild inner steel tube reinforcement within concrete-filled weathering steel tubes, evaluated through lateral impact loading tests demonstrating substantial resistance to lateral impacts. Their research involved developing numerical models to validate experimental outcomes and conducting parametric analyses to optimize design parameters. Niknejad et al. [15] investigated the energy absorption capabilities of circular polyurethane foam-filled composite tubes under lateral compression. Load–displacement and energy–displacement diagrams were produced, and energy absorption per unit mass was determined. This study examined the effects of geometrical characteristics, foam-composite tube adhesion, and foam density. Comparisons were made between the deformation modes of empty and foam-filled tubes. It was concluded that longer foam-filled composite tubes with additional fiber fabric layers demonstrated superior energy absorption and more orderly deformation. The flattening of foam-filled E-glass/vinylester composite tubes was identified as an effective energy absorption mechanism. Moeinifard et al. [16] investigated unstiffened and grid-stiffened composite cylindrical shells under compression, utilizing two loading configurations: compression between rigid platens and via a cylindrical indenter perpendicular to the specimen axis and a rigid platen. Their findings indicated a significant increase in energy absorption capability for grid-stiffened shells, surpassing that of unstiffened variants. Despite extensive studies on the lateral behavior of composite tubes and investigations into various mechanical and geometrical parameters affecting their performance and failure mechanisms [17,18,19,20,21,22,23,24,25], gaps persist in understanding the mechanical performance of different circular and semicircular composite sections during the indentation flattening process. Further research is essential to comprehensively explore how these sections respond to lateral indentation compressive load and the factors influencing their structural integrity and energy absorption capabilities. This study addresses this gap through experimental investigations on cylindrical and semicircular composite tubes filled with polyurethane foam subjected to indentation by various parallel steel rods. In this paper, a deeper investigation is conducted into the crashworthiness behavior and energy absorption capacity of composite tubes under lateral indentation by steel rods. Building on previous findings, key parameters such as tube diameter, length, wall thickness, and indenter diameter are examined, and their effects on the mechanical properties and structural response of both semicircular and circular composites are compared. Additionally, the influence of polyurethane foam fillers on damage modes and energy absorption capacity is rigorously analyzed, with a focus on the complex interplay of damage mechanisms like matrix cracking, fiber breakage, foam crushing, foam densification, foam fracture, and debonding of composite layers. By exploring these factors, optimal configurations that maximize energy absorption and improve crashworthiness are identified. Ultimately, valuable insights are provided to inform the design and engineering of more efficient and resilient composite structures.

## 2. Experimental Procedure

### Materials and Methods

**Figure 1:**
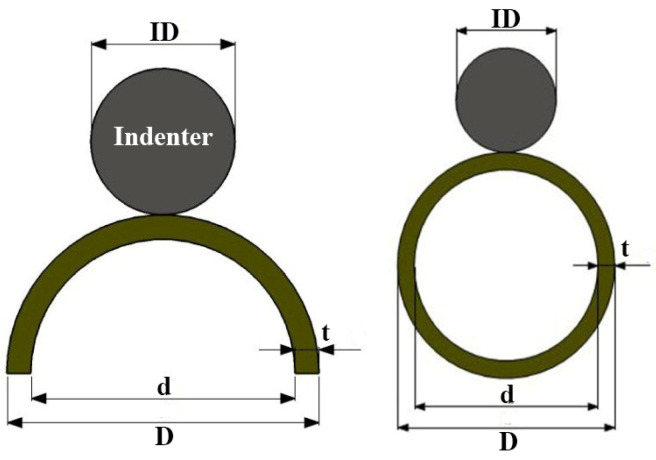
Representation of indentation procedures on semicircular (**left**) and cylindrical (**right**) composite tubes.

**Figure 2:**
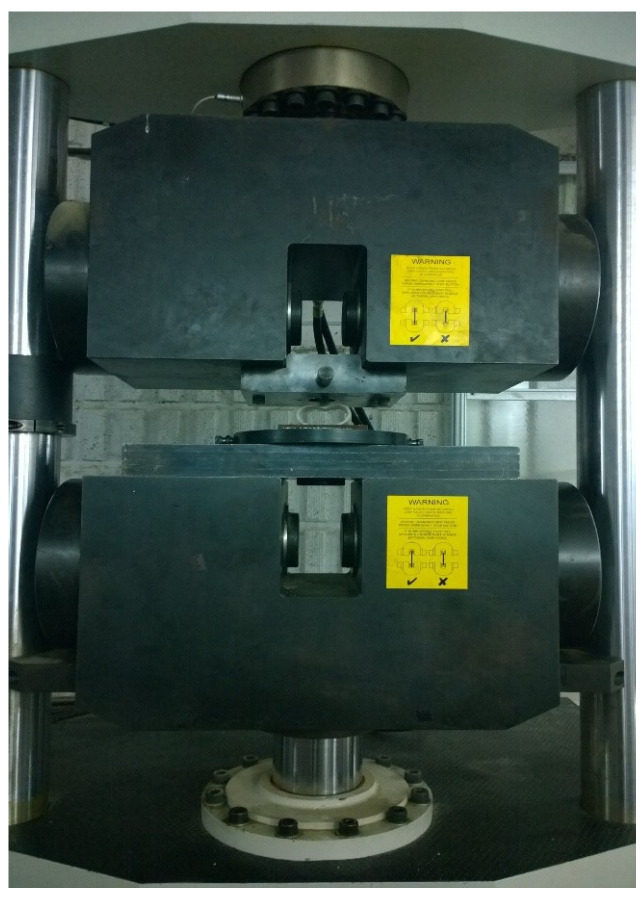
Setup for lateral indentation test of composite specimen B-04.

Glass/epoxy composite tubes, infused with polyurethane foam and woven in a +60/−60 configuration, were subjected to quasi-static compression utilizing rigid cylindrical indenters aligned parallel to the specimen axis. The specimens were meticulously manufactured and divided into three categories: semicircular foam-filled tubes; empty cylindrical tubes; and foam-filled cylindrical tubes. The geometrical dimensions of the empty and foam-filled composite cylindrical specimens, along with indenter diameters (ID), are presented in Table 1, whereas Table 2 details the dimensions for the foam-filled semicircular specimens. For each group, multiple specimens with varying lengths (L), diameters (d), and thicknesses (t) were prepared and tested, as depicted in Figure 1. Throughout all experiments, the crosshead speed was consistently maintained at 2 mm/min. The polyurethane foam employed had a density of 256 kg/m^3^ and a plateau stress of 1.76 MPa. Polyurethane foam is produced through molecular interactions between polyol and isocyanate components. Achieving the desired foam characteristics necessitates specific reaction conditions and a precise mix. Polyol contributes flexibility and resilience to the foam, while isocyanate serves as a curing agent, enhancing the foam’s strength and stiffness. Each test was repeated three times to ensure the accuracy and consistency of the results. Figure 2 illustrates an empty specimen during the indentation test conducted using a DMG tensile testing machine. In all lateral indentation tests, graphs depicting lateral load versus lateral displacement and absorbed energy per unit length of the composite specimen versus displacement were generated. Furthermore, total energy absorption capacity and specific absorbed energy were calculated and analyzed.

## 3. Results and Discussion

The impact of various geometrical dimensions on both the contact force and the energy absorption capability of the specimens under examination is detailed in the subsequent sections. This investigation explores how dimensions such as indenter diameter, tube length, diameter, and thickness influence these critical performance metrics.

### 3.1. Effects of Tube Length

**Figure 3:**
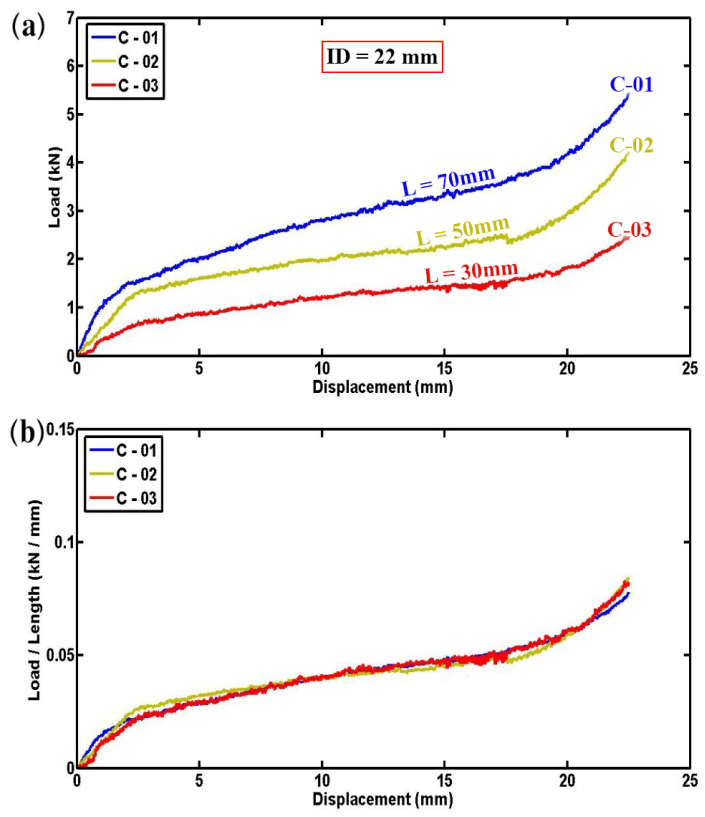
(**a**) Load versus displacement plots and (**b**) Load normalized by length–displacement diagrams for foam-filled semicircular composite tubes having different lengths (d = 61.4 mm, t = 1.3 mm, ID = 22 mm).

**Figure 4:**
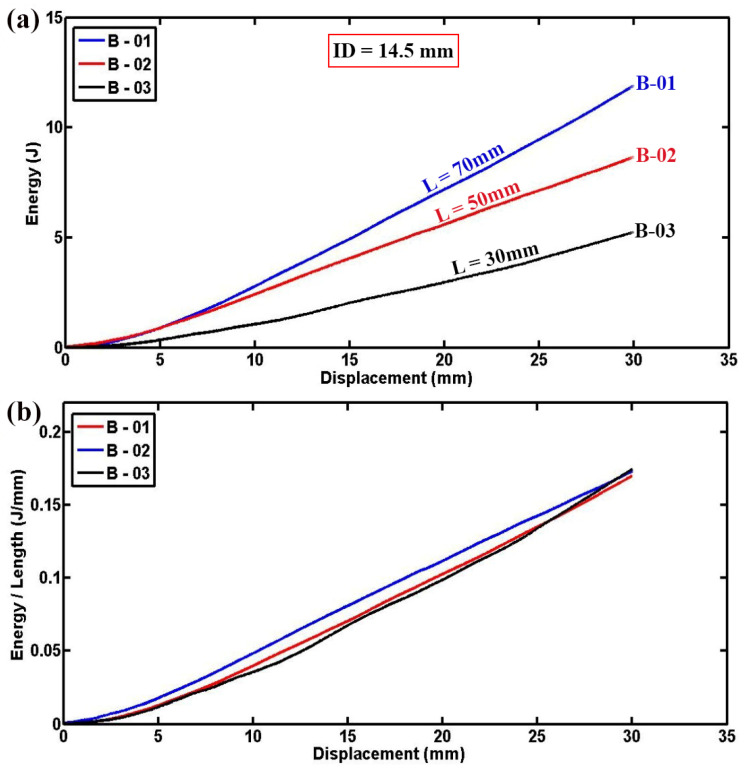
(**a**) Energy–displacement and (**b**) Energy per unit length–displacement curves of empty cylindrical specimens sharing the same diameter and thickness but differing in length (d = 50 mm, t = 2.2 mm, ID = 14.5 mm).

Figure 3a presents the variation in lateral load versus displacement for foam-filled semicircular specimens, illustrating a clear trend where the lateral contact force increases in a nearly linear fashion with the length of the tube. Fluctuations in the load–displacement curves are observed due to progressive failure mechanisms, including the breaking of fibers, the cracking of the matrix, and the crushing of foam in the area where the specimens are contacted by the indenter. To mitigate the influence of tube length on the results, Figure 3b provides normalized lateral load–displacement diagrams for foam-filled specimens C-01, C-02, and C-03. This normalization reveals consistent compressive contact forces per unit length across specimens C-01, C-02, and C-03 despite their varying overall lengths but identical geometric dimensions. Figure 4a,b further elaborates on the experimental outcomes by presenting the instantaneous absorbed energy and absorbed energy per unit length for three different empty specimens of varying lengths. As depicted in Figure 4a, there is a consistent and proportional increase in absorbed energy with the length of the tube reinforcing the reliability and repeatability of the experimental setup. The similarity observed in the curves shown in Figure 4b further affirms the accuracy of the experimental results, indicating a robust correlation between tube length and energy absorption capability.

**Figure 5:**
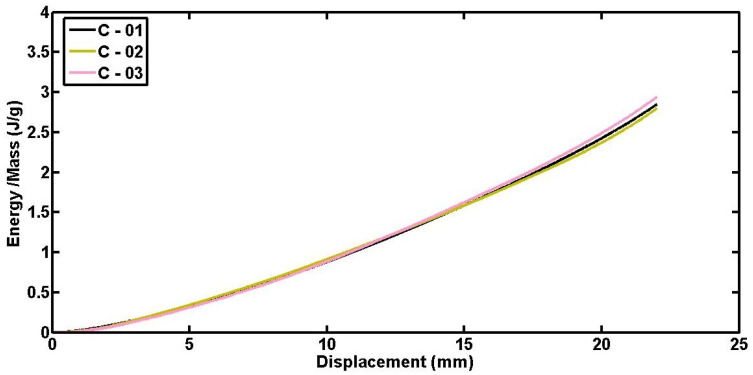
Specific absorbed energy variation with displacement for specimens having consistent diameter and thickness but differing lengths (d = 61.4 mm, t = 1.3 mm, ID = 22 mm).

To delve deeper into the influence of tube length and to validate the experimental precision, Figure 5 explores the specific absorbed energy (SAE) of foam-filled semicircular tubes (C-01, C-02, and C-03) plotted versus lateral displacement. Remarkably, Figure 5 illustrates that nearly identical specific absorbed energy values are exhibited by all three foam-filled semicircular tubes, highlighting the high precision in both the preparation and testing of the specimens. Furthermore, a direct relationship between the tube length and both load capacity and specific absorbed energy is observed. This consistency across specimens reaffirms the reliability of the experimental setup and enhances confidence in the obtained results regarding energy absorption capabilities under lateral displacement conditions.

### 3.2. Effects of Polyurethane Foam

**Figure 6:**
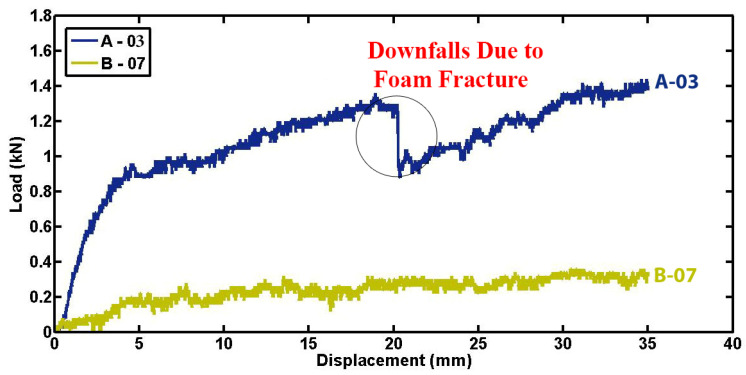
Load–displacement curves for foam-filled and empty specimens of matched characteristics and indenter diameter (d = 60.4 mm, t = 1.8 mm, L = 50 mm, ID = 22 mm).

**Figure 7:**
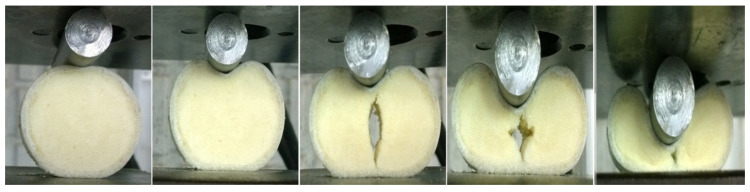
Visual depiction of foam failure in specimen A-03 under lateral compression.

Figure 6 illustrates the relationship between compression load and lateral displacement for foam-filled specimen A-03 and empty specimen B-07, both sharing identical dimensions in terms of length, diameter, thickness, and indenter diameter. The figure highlights the substantial impact of the polyurethane foam filler on the contact force and, consequently, on the energy absorption capabilities of the foam-filled specimen compared to the empty one. In quasi-static lateral compression tests, the load-carrying capacity of composite tubes is notably increased by the incorporation of polyurethane foam. The foam ensures a more uniform distribution of the applied load, which reduces stress points and prevents early failure at the interface between the composite material and the indenter. By deforming, the foam absorbs and dissipates energy, functioning as a crushable layer that progressively takes on the load. Moreover, the foam enhances the overall stiffness of the composite tube, making it more resistant to both bending and deformation. The indentation process of specimen A-03 is further detailed in Figure 7. During the lateral indentation test, a sequence of damage mechanisms occurs, progressively enhancing energy absorption. Initially, the indenter contacts the specimen, causing elastic deformation. As loading continues, matrix cracking begins, followed by fiber breakage and delamination at the contact area. Further away, plastic hinges form due to plastic deformation. With increased load, the foam core undergoes crushing and subsequent densification, absorbing significant energy. Notably, this figure shows that after approximately 20 mm of displacement, a crack initiates within the foam and propagates as the specimen undergoes quasi-static compression. This phenomenon occurs due to the development of indirect tensile stresses within the foam under specific loading conditions, causing the crack to propagate perpendicular to the direction of maximum principal stress. The observed fracture of the foam in the filled specimen correlates with the decline in load observed in the load–displacement curve of specimen A-03 in Figure 6.

**Figure 8:**
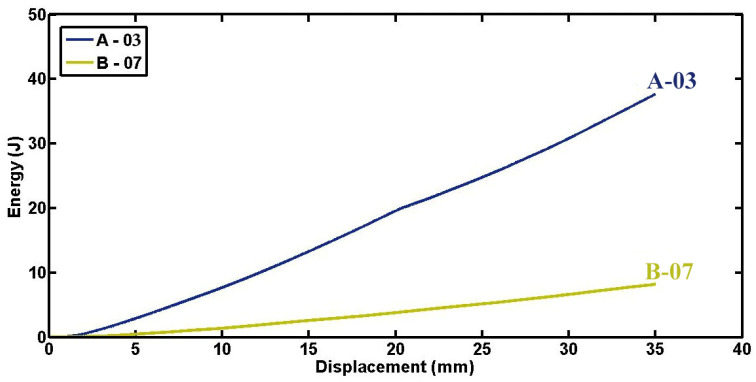
Comparison of energy absorption–displacement diagrams between foam-filled specimen A-03 and empty specimen B-07 having identical geometrical characteristics (d = 60.4 mm, t = 1.8 mm, L = 50 mm, ID = 22 mm).

Figure 8 illustrates the relationship between energy absorption and displacement for foam-filled cylindrical specimen A-03 and empty specimen B-07, both characterized by identical geometrical parameters and subjected to equivalent loading conditions. The graph shows that up to a displacement of 35 mm, specimen A-03 absorbed 37.7 J of energy, whereas specimen B-07 absorbed only 8.2 J. This substantial disparity highlights the pronounced influence of polyurethane foam as a lightweight material possessing exceptional energy absorption capabilities. Specifically, the specific energy absorptions of specimens A-03 and B-07 up to 35 mm displacement are computed as 618.65 J/kg and 330.78 J/kg, respectively. This comparison underscores the superior energy absorption efficiency conferred by polyurethane foam compared to an empty structure under analogous compression scenarios.

**Figure 9:**
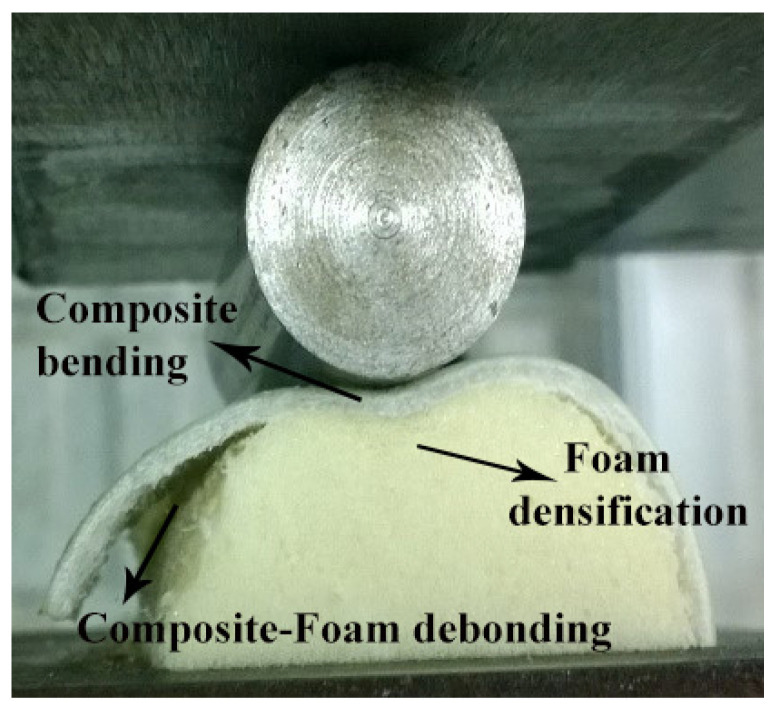
The interface debonding in the semicircular composite tube and foam induced by circumferential bending (C-05).

During the indentation process shown in Figure 9, debonding between the composite inner wall and the polyurethane foam in the foam-filled semicircular specimens was observed. This phenomenon is attributed to the bending moment generated within the semicircular specimen during lateral indentation, influenced by the presence of foam. The interaction between the foam and the composite material introduces stress concentrations and shear forces at the interface, potentially exceeding the bonding strength and leading to debonding. Additionally, the foam’s deformation under load can exacerbate this effect, particularly if the adhesive bond is inadequate. Differences in mechanical properties between the composite and foam, such as stiffness and thermal expansion coefficients, further contribute to stress concentration and differential movement, ultimately promoting debonding.

### 3.3. Effects of Tube Diameter

**Figure 10:**
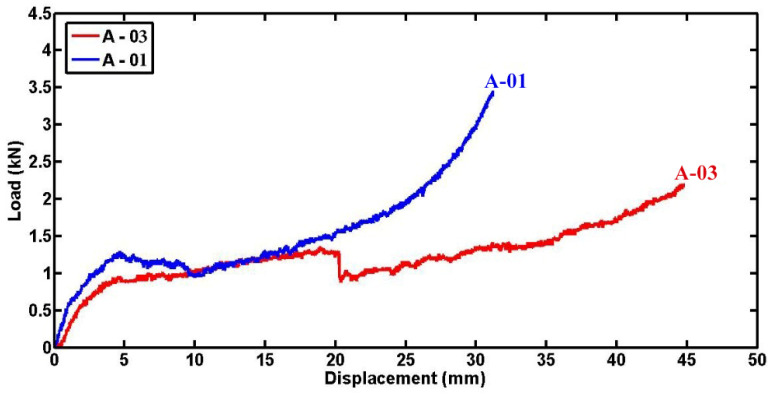
Load–displacement diagrams comparing filled cylindrical specimens with uniform length, thickness, and indenter diameter but differing tube diameters (L = 50 mm, t = 1.8 mm, and ID = 22 mm).

To explore the influence of tube diameter, Figure 10 presents lateral compression load–displacement diagrams for two filled cylindrical specimens. These specimens share identical length, thickness, and indenter diameter but differ in tube diameter, maintaining a constant displacement-to-diameter ratio of 0.7. Up to a displacement of 15 mm, both specimens exhibit comparable contact forces. However, beyond 15 mm displacement, the specimen with the smaller diameter demonstrates a higher ability to withstand lateral force compared to its larger diameter counterpart. This observation underscores the significant impact of tube diameter on the mechanical response during lateral compression testing. The higher elastic load slope observed in foam-filled composite tubes with smaller diameters can be attributed to their inherently greater stiffness due to the reduced cross-sectional area. This increased stiffness enables them to resist deformation more effectively under applied loads.

**Figure 11:**
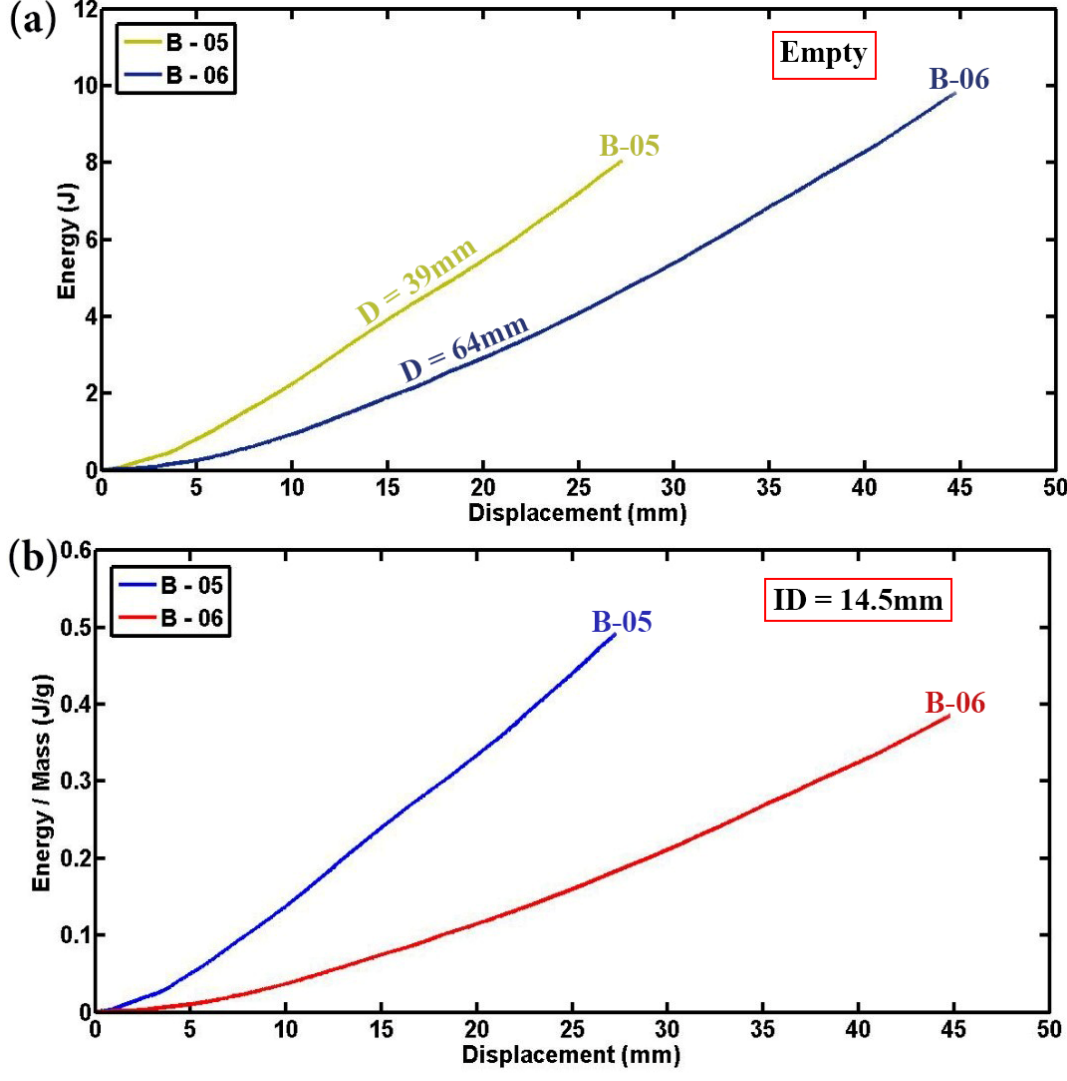
Analysis of tube diameter influence on (**a**) energy displacement and (**b**) energy per unit mass–displacement patterns of two empty specimens (L = 50 mm, t = 1.8 mm, ID = 14.5 mm).

**Figure 12:**
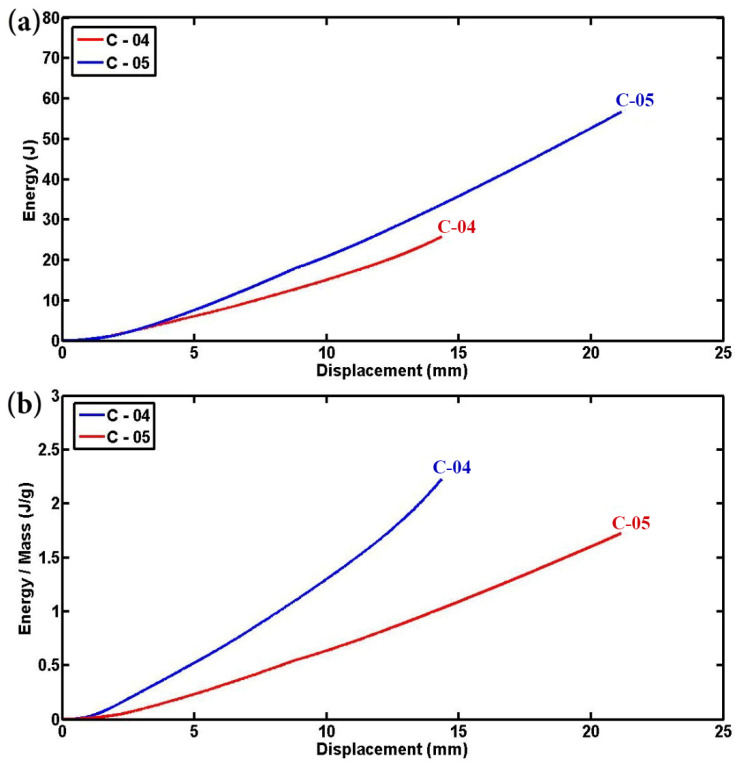
Comparison of tube diameter effects on the (**a**) energy and (**b**) specific energy absorption in two semicircular foam-filled composite specimens (L = 50 mm, t = 1.8 mm, and ID = 14.5 mm).

For a comprehensive investigation into the influence of tube diameter, Figure 11 and Figure 12 provide detailed insights into the absorbed energy and specific absorbed energy of empty and foam-filled specimens, respectively. Figure 11a,b clearly demonstrates that up to the same displacement-to-diameter ratio, specimens with smaller diameters absorb less total energy but exhibit higher specific energy absorption compared to those with larger diameters. This observation aligns with established research, which indicates that smaller-diameter empty specimens have superior performance due to higher contact force, lower total energy absorption, and greater specific energy absorption. Conversely, foam-filled specimens with larger diameters absorb more total energy but have lower specific energy absorption compared to their smaller diameter counterparts, as the increased volume of foam in larger tubes facilitates greater overall energy absorption but less efficient absorption per unit volume. Notably, this study employs a cylindrical indenter rather than a rigid flat platen for specimen indentation, differing from previous methodologies [6,15], which affects the observed mechanical responses. The findings reaffirm that empty cylindrical specimens with smaller diameters consistently exhibit higher contact force, lower energy absorption, and greater specific energy absorption, supporting earlier conclusions. Additionally, foam-filled cylindrical and semicircular specimens with smaller diameters show enhanced mechanical performance, characterized by higher contact force and specific absorbed energy, due to improved stress concentration and effective engagement of polyurethane foam within the smaller tubes.

### 3.4. Effects of Tube Thickness

**Figure 13:**
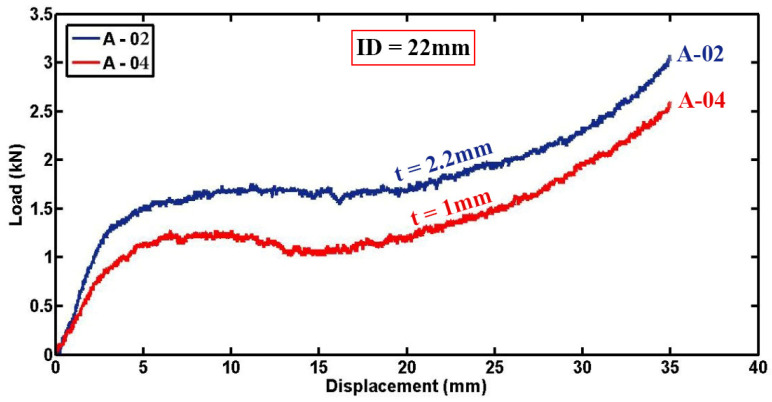
Load versus displacement diagrams for foam-filled specimens of different thicknesses but identical geometrical parameters (D = 54.4 mm, L = 50 mm, ID = 22 mm).

The load–displacement plots of two foam-filled specimens (A-02 and A-04) with identical lengths, tube inner diameter, and indenter diameter but varying thicknesses are depicted in Figure 13. The increase in indentation forces with increasing specimen thickness can be attributed to the greater structural stiffness inherent in thicker specimens. Thicker materials typically offer higher resistance to deformation under applied loads, resulting in lateral loads of 1.6 kN and 1.08 kN observed for specimens A-02 and A-04, respectively, at 15 mm displacement. This trend underscores the direct relationship between specimen thickness and lateral load capacity, emphasizing the influence of material thickness on the mechanical response during lateral compression testing.

**Figure 14:**
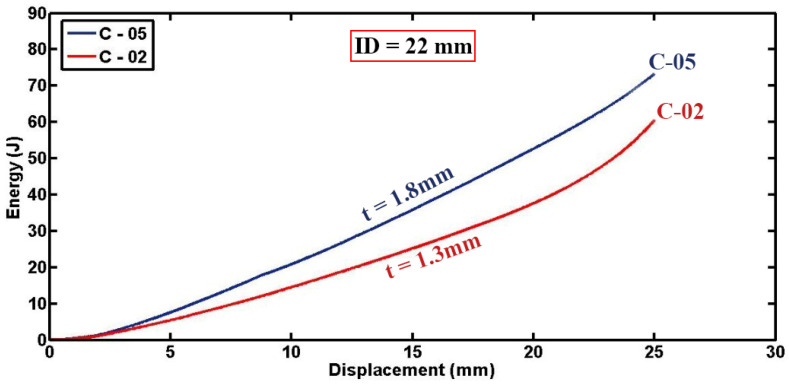
Energy absorption versus displacement curves for specimens of different thicknesses (D = 64 mm, L = 50 mm, ID = 14.5 mm).

Furthermore, Figure 14 provides diagrams, plotting absorbed energy versus lateral displacement for two different configurations of foam-filled semicircular specimens. The figures illustrate that increasing the thickness of the semicircular tubes leads to higher levels of energy absorption. For instance, the thicker specimen C-05 absorbed 35.83 J of energy, while the sample C-02, which had lower thickness but identical geometric dimensions, absorbed 25.16 J of energy, up to a displacement of 15 mm. This trend can be justified by the greater volume of material available for energy dissipation in thicker tubes, resulting in enhanced crashworthiness performance. The direct relationship observed between tube thickness and energy absorption capacity underscores the critical role of geometrical parameters in optimizing the mechanical response and structural integrity of composite materials under lateral indentation loading conditions.

### 3.5. Effects of Indenter Diameter

**Figure 15:**
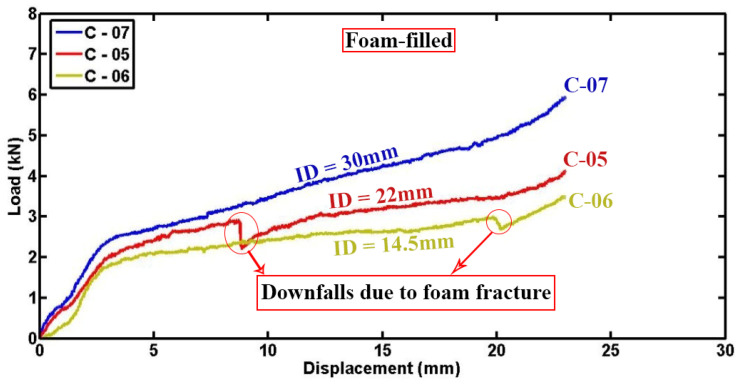
Indenter diameter influence on load–displacement profiles of foam-filled semicircular specimens (d = 60.4 mm, L = 50 mm, t = 1.8 mm).

**Figure 16:**
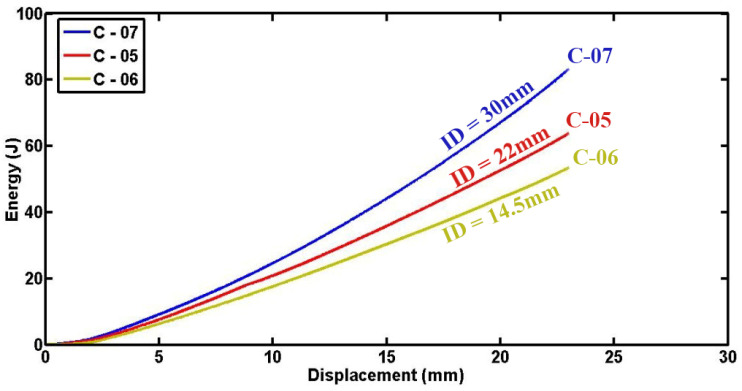
Comparison of absorbed energy at varying displacements among specimens with different indenter diameters (d = 60.4 mm, L = 50 mm, t = 1.8 mm).

Figure 15 and Figure 16 depict detailed plots of compressive load and absorbed energy versus lateral displacement for foam-filled semicircular specimens with the same geometries tested under varying indenters. It is evident from the data that increasing the diameter of the indenter is associated with higher compressive forces and greater absorbed energy. This correlation can be scientifically explained by the mechanical interactions involved: firstly, the larger diameter of the indenter results in a larger contact area with the specimen, allowing for more extensive crushing and deformation of the polyurethane foam, thereby enabling greater participation of the foam material in energy absorption and dissipation. Secondly, the higher localized stresses induced by the larger indenter diameter promote more efficient energy absorption mechanisms through enhanced foam deformation. Moreover, it should be noted that fluctuations in the load–displacement diagrams can be attributed to progressive damage mechanisms inherent in composite materials, including fiber breakage, matrix cracking, and foam fracture. Specifically, specimens C-05 and C-06 may experience sudden drops in load due to foam fracture under higher compressive forces, resulting in abrupt decreases in the load–displacement curves. These observations underscore the dynamic and complex nature of energy absorption in composite structures under lateral indentation loading conditions, where the overall performance and durability are significantly influenced by the interplay between material properties, geometrical parameters like indenter diameter, and damage mechanisms.

**Figure 17:**
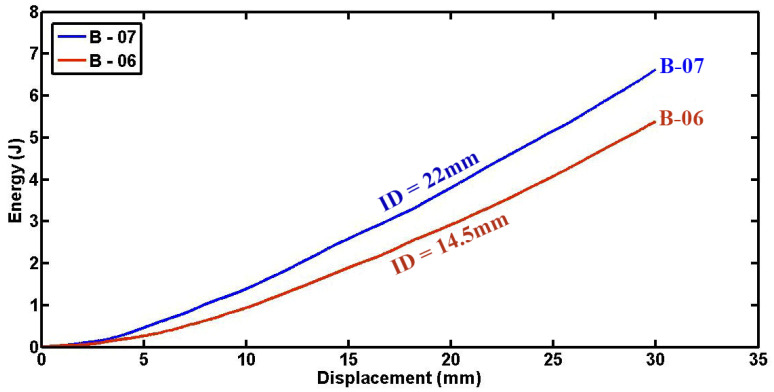
Absorbed energy as a function of displacement in specimens of uniform dimensions but differing tube diameters (d = 60.4 mm, L = 50 mm, t = 1.8 mm).

Figure 17 illustrates the impact of indenter diameter on the absorbed energy of empty cylindrical specimens. These data indicate a notable increase in absorbed energy with larger indenter diameters. This trend mirrors observations made in foam-filled cylindrical specimens as well. The larger indenter diameter allows for greater deformation and engagement of the specimen material, particularly significant in enhancing energy absorption capabilities during compression tests. Thus, indenter diameter emerges as a critical parameter influencing the energy absorption performance of both empty and foam-filled cylindrical specimens under quasi-static loading conditions.

**Figure 18:**
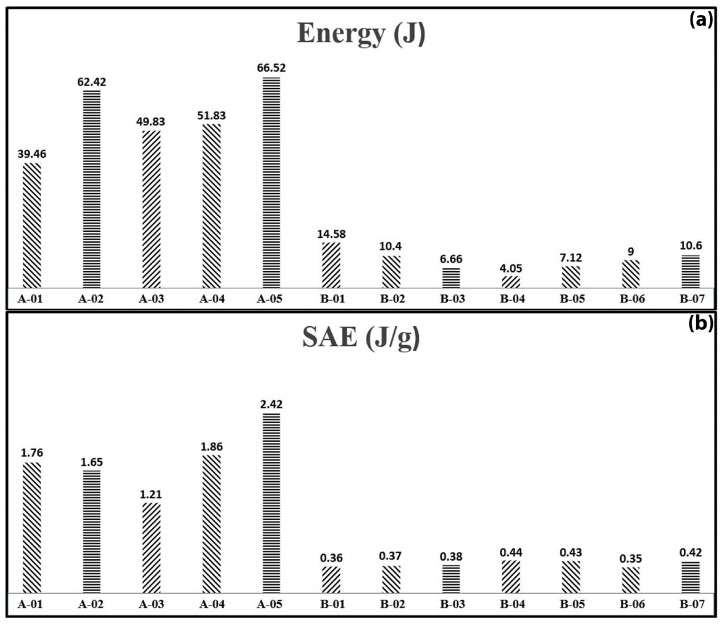
Analysis of absorbed energy (**a**) and specific absorbed energy (**b**) in the tested cylindrical specimens.

**Figure 19:**
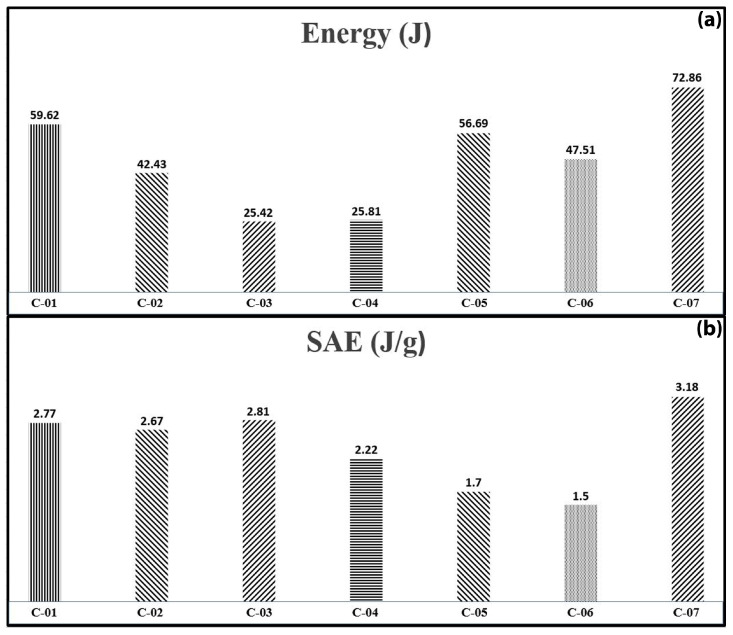
Absorbed energy (**a**) and specific absorbed energy (**b**) variation across tested semicircular specimens.

To provide a thorough comparison among specimens with different geometrical dimensions, Figure 18 and Figure 19 display the absorbed energy and specific absorbed energy for both cylindrical and semicircular specimens. These figures are plotted based on consistent displacement-to-diameter ratios of 0.7 and 0.35, respectively, to standardize the evaluation of energy absorption characteristics across various specimen types and geometries. The bar charts in Figure 18 show that foam-filled specimens (A series) outperform empty composite tubes (B series) in both total energy absorption and specific absorbed energy under lateral indentation. Specimen A-05 is particularly notable for its efficiency, which is mainly attributed to its larger indenter diameter of 30 mm compared to the 22 mm diameter used for other A series specimens. The B series has significantly lower energy absorption, with B-01 absorbing the most energy at 14.58 J and B-04 the least at 4.05 J, indicating that empty composite tubes are less effective in energy absorption compared to foam-filled specimens. Figure 19 shows the absorbed energy and specific absorbed energy variations for semicircular specimens C-01 to C-07. Specimen C-07 performs the best, with the highest absorbed energy at 72.86 J and the highest specific absorbed energy (SAE) at 3.18 J/g, indicating its superior efficiency in energy absorption relative to its mass. Specimens C-01, C-05, and C-06 also show significant energy absorption, although C-06 has the lowest SAE at 1.5 J/g, highlighting its relatively lower efficiency. Specimens C-03 and C-04 have the lowest energy absorption, around 25 J, with C-03 performing better in terms of SAE at 2.81 J/g. The bar charts demonstrate that polyurethane foam filler significantly enhances the energy absorption capacity of composite sections under lateral indentation, outperforming other parameters.

## 4. Conclusions

This study experimentally investigated the energy absorption capacity and crashworthiness behavior of polyurethane foam-filled composite cylindrical and semicircular tubes. Various specimens differing in length, thickness, and diameter were subjected to lateral indentation using different indenters at a constant quasi-static loading rate. The results indicated that increased tube thickness, length, and indenter diameter, alongside decreased tube diameter, correlated with higher contact forces and improved energy absorption. Notably, larger indenter diameters significantly enhanced compressive forces and energy absorption in both foam-filled and empty cylindrical specimens due to the larger contact area, as well as allowing for more extensive crushing and deformation of the polyurethane foam. This underscores the indenter diameter’s critical role in optimizing energy absorption capabilities under lateral loading conditions. The composite specimens exhibited complex damage mechanisms, including matrix cracking, fiber breakage, foam crushing, foam densification, foam fracture, and debonding of composite layers. These progressive damage modes collectively influenced the contact force–displacement curves and significantly impacted energy absorption capacities. Contrary to conventional expectations from studies on empty or rigidly compressed tubes, smaller diameter foam-filled tubes demonstrated superior crashworthiness properties, characterized by higher contact forces and specific absorbed energies. This finding highlights the importance of foam behavior and composite damage mechanisms in shaping the overall energy absorption process. A comprehensive understanding of these mechanisms is essential for optimizing the design of composite structures to enhance crashworthiness across diverse engineering applications.

## Figures and Tables

**Table 1:** Geometrical details of composite cylindrical tubes (empty and foam-filled).

Part No.	D (mm)	d (mm)	t (mm)	L (mm)	M (g)	ID (mm)	Tube Type
A-01	44.6	41	1.8	50	22.4	22	Filled
A-02	54.4	50	2.2	50	37.74	22	Filled
A-03	64	60.4	1.8	50	41.04	22	Filled
A-04	54.4	52.4	1	50	27.8	22	Filled
A-05	54.4	52.4	1	50	27.41	30	Filled
B-01	54.4	50	2.2	70	40.34	14.5	Empty
B-02	54.4	50	2.2	50	27.71	14.5	Empty
B-03	54.4	50	2.2	30	17.38	14.5	Empty
B-04	64	61.4	1.3	50	9.15	14.5	Empty
B-05	39	35.4	1.8	50	16.35	14.5	Empty
B-06	64	60.4	1.8	50	25.5	14.5	Empty
B-07	64	60.4	1.8	50	25.55	22	Empty

**Table 2:** Characteristics and indenter sizes for foam-filled composite semicircular tubes.

Part No.	D (mm)	d (mm)	t (mm)	L (mm)	M (g)	ID (mm)
C-01	64	61.4	1.3	70	21.84	22
C-02	64	61.4	1.3	50	15.87	22
C-03	64	61.4	1.3	30	9.03	22
C-04	44.6	41	1.8	50	11.6	22
C-05	64	60.4	1.8	50	32.88	22
C-06	64	60.4	1.8	50	31.54	14.5
C-07	64	60.4	1.8	50	31.84	30

## Data Availability

The original contributions presented in this study are included in this article; further inquiries can be directed to the corresponding author.
